# Wild poliovirus case in Malawi reinforces need to continue eradication efforts

**DOI:** 10.2471/BLT.22.020622

**Published:** 2022-06-01

## Abstract

Despite recent challenges, polio eradication is tantalizingly close. Gary Humphreys reports.

Since someone stole her motorcycle, Mammie Moyo has been doing a lot of walking. A senior health surveillance assistant at the Enukweni Health Centre in Mzimba, Northern Malawi, Moyo distributes oral polio vaccine to villages in the surrounding area, walking up to 26 km on some days, an insulated vaccine carrier on her shoulder.

“It is hard, but we do it for the children,” says the 46-year-old mother of two, who is one of 13 500 health workers who have been trained to vaccinate Malawi’s children.

Like most people in her community, Moyo was shocked by the news of a young girl in Malawi falling ill with poliomyelitis in November 2021.

The 3-year-old girl – who lives in the capital, Lilongwe – was diagnosed with acute flaccid paralysis (AFP), a syndrome characterized by the abrupt onset of limb weakness or paralysis with reduced muscle tone. Specimens of her stool were tested a week later, and wild poliovirus identified.

Prior to the Lilongwe incident, it had been widely assumed that wild poliovirus was no longer circulating outside of Afghanistan and Pakistan.

“Malawi came as a shock,” says Mike McGovern, Chair of Rotary's international PolioPlus programme. Rotary has been working on polio eradication since the mid-1980s and with the Bill & Melinda Gates Foundation has raised around 2 billion United States dollars (US$) in funding and mobilized a further US$ 8 billion from the public sector. “It was the first case in Malawi since 2016 and a reminder of the often-repeated fact that polio anywhere is polio everywhere.”

Genetic analysis done in February 2022 showed that the virus infecting the little girl was closely related to a strain detected in a sewage sample from northern Sindh, in Pakistan, in October 2019.

Somehow the wild virus had travelled between two locations separated by the Arabian Peninsula, dozens of countries and around 12 000 km of road.

“How and over what period of time the virus made its journey is clearly a major source of concern for us,” says Aidan O’Leary, director of the Global Polio Eradication Initiative (GPEI), which was set up in 1988, after the World Health Assembly passed a resolution calling for the eradication of the disease.

In the worst-case scenario, the virus has been slowly spreading with the movement of populations.

“Malawi came as a shock.”

Mike McGovern

Many in Lilongwe, a city with a population of roughly 1 million people, have significant contact with people in neighbouring countries including United Republic of Tanzania and Zambia, who in turn connect with people in Ethiopia and Somalia and countries across the Horn of Africa.

But if the virus had been making its way across land, it might have been expected to appear along the way, especially given the surveillance capacity in many countries, notable among them Afghanistan and Pakistan.

However, surveillance has not been maintained at the same level everywhere. As the disease has retreated, countries have deprioritized such efforts, Malawi included – winding down polio surveillance after Africa was declared free of wild poliovirus in 2016.

Moreover, despite its devastating impact on some children, polio is not very easy to track. Only one in every 200 children is paralysed, which means that a significant number can contract the disease and pass it on without ever knowing it. Adding to the surveillance challenge is the fact that AFP, the default indicator for potential polio infection, can be caused by other infectious and non-infectious diseases.

To track polio incidence and prevalence, surveillance teams must investigate every reported case of AFP. “Active case finding is labour-intensive, demanding work, but it really is the gold standard,” explains Dr Mandeep Rathee, Deputy Team Leader for the World Health Organization’s (WHO) polio programme in Afghanistan, where some 4000 cases of AFP were investigated in 2021, poliovirus being isolated in just one stool sample.

Since the Lilongwe case, surveillance has been stepped up by health authorities in Malawi, backed by a GPEI Rapid Response Team. As of late April, surveillance teams had taken faecal samples from 72 children presenting with AFP without isolating poliovirus in either its wild or vaccine-derived forms.

Environmental sampling – the other mainstay of polio surveillance – has also been ramped up in Malawi, with eight surveillance sites set up in Lilongwe’s sewers. None of the 54 environmental samples collected by the end of April 2022 contained poliovirus.

According to O’Leary, surveillance is also being increased in neighbouring countries Mozambique, United Republic of Tanzania, Zambia and Zimbabwe, and is being reviewed in Afghanistan and Pakistan where AFP investigation and extensive environmental surveillance systems are in place, and have been maintained despite challenges on the ground.

It is hoped that these efforts may shed some light on the journey made by the Lilongwe virus, but as O’Leary points out, at this time there are more questions than answers. “What we have to do is keep testing, testing and testing,” he says.

And vaccinating – the other core activity of the global polio response. Here too, the authorities in Malawi, supported by their international partners, have been busy. The Government of Malawi declared a Public Health Emergency on 17 February 2022 and launched a response plan that includes at least four rounds of nationwide supplementary immunization campaigns using an oral poliovirus vaccine.

The first two rounds were conducted in March and April, implemented by the Government of Malawi with the support of GPEI partners including the United Nations Children’s Fund (UNICEF), WHO and local Rotarians. By the end of April just under 3 million children had been vaccinated.

WHO recommends that countries with endemic polio, or where the risk of imported cases is high, implement oral poliovirus vaccination at birth followed by a primary series of three oral doses and at least one dose with inactivated polio vaccine (an injectable vaccine), starting at 6 weeks of age.

Global, routine polio immunization has declined in recent years as the coronavirus disease 2019 (COVID-19) pandemic and associated disruptions have strained health systems, with 83% immunization coverage among 1-year-olds in 2020 down from 86% in 2019.

Unrest has also taken a toll, notably in Afghanistan where house-to-house vaccination campaigns were suspended in 2018 in areas controlled by the Taliban, and 3 million children missed routine vaccination. The near-collapse of the Sehatmandi-supported health system in 2021 further exacerbated the challenges faced.

However, in October of 2021, the Taliban agreed to allow health workers to begin a nationwide polio vaccination campaign, including house-to-house vaccination. According to Rathee, 9.8 million children were vaccinated in the most recent nationwide polio campaign which concluded in March 2022, representing assessed coverage of around 86%.

“There is clearly an opportunity to bring this home.”

Aidan O’Leary

For O’Leary, maintaining elevated vaccination rates (90% or above) is not only vital to eradicating polio – it is also key to tackling one of the niggling challenges of the polio end game, namely vaccine-derived polio infection.

Vaccine-derived polio occurs when the attenuated virus used in oral polio vaccines replicates in the intestine and is excreted. In places where sanitation and access to clean water is lacking and the local population is under-immunized, the attenuated virus can pass through multiple human hosts and over 12 to 18 months adapt and reacquire neurovirulence.

Vaccine-derived poliovirus infection is on the rise, with 1074 cases confirmed in 24 mostly African countries in 2020, compared with 366 cases from 16 countries in 2019. To address the problem, GPEI partners are working to deploy a new oral polio vaccine – novel oral polio vaccine type 2 – which is genetically more stable and less likely to reacquire neurovirulence.

According to O’Leary, there are early indications that vaccine-derived polio infection cases fell in 2021 and, overall, he remains positive. “Vaccine-derived polio strains are clearly a concern, but by intensifying their vaccination efforts, countries can reduce the risk of such strains arising,” he says.

He is also keen to point out how dramatically the number of wild poliovirus cases has fallen in the past 2 years. “We've gone from 140 cases in 2020 to just six in 2021 – four in Afghanistan, one in Pakistan, and one in Malawi. So far this year, we have seen just one case in Afghanistan and two cases in Pakistan. There is clearly an opportunity to bring this home,” he says.

One possible reason for the drop in cases is the COVID-19 pandemic, which has hampered polio eradication efforts but has also thrown up obstacles to transmission. “Reduced mobility – both within Afghanistan and Pakistan, and across the borders, may have had an impact,” says Rathee. “There has also been a change in social norms, with very simple things like social distancing and hand washing.”

Another reason is the sheer grit and determination of people like Mammie Moyo, who, despite losing her motorbike, trudges 26 km through the forest with her vaccine pack to make sure that children get the vaccine they need.

“When they heard about the girl in Lilongwe everyone was shocked,” she says. “Everyone thought polio was something from the past. I will keep walking until that is true.”

**Figure:**
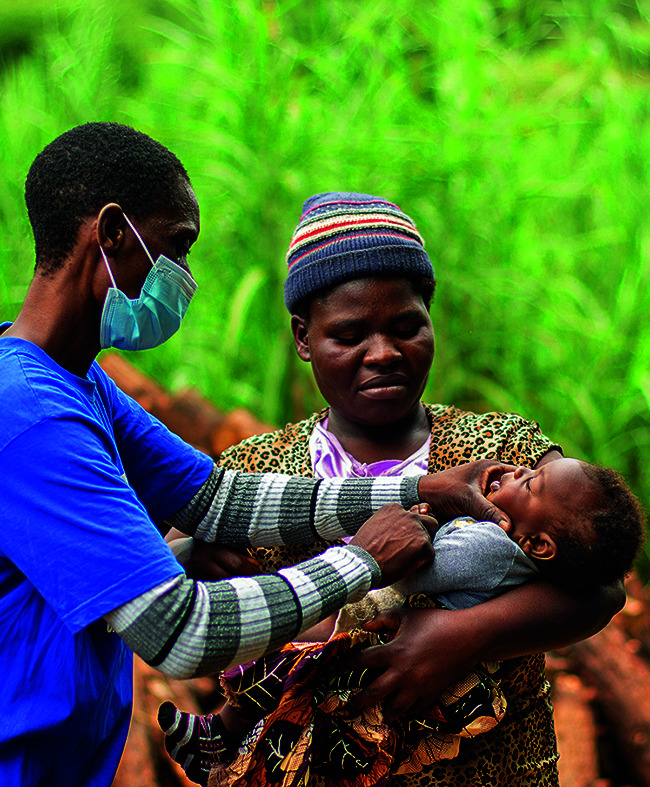
Mammie Moyo administers oral polio vaccine to an infant in Mzimba, Northern Malawi

UNICEF/UN0616336

**Figure:**
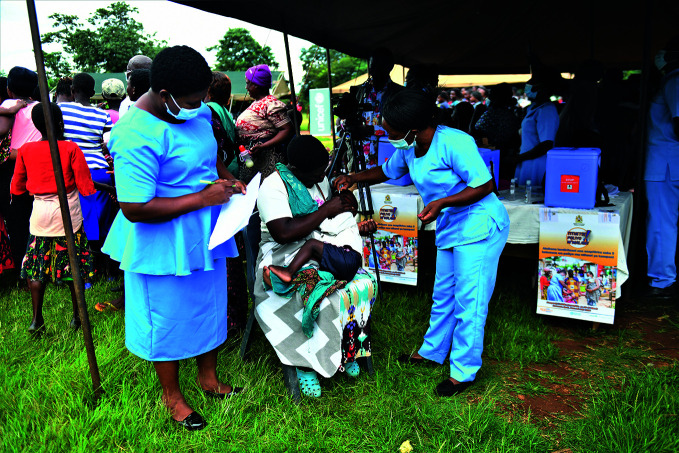
Health workers administer oral polio vaccine at a temporary vaccination centre in Northern Malawi

UNICEF/Thoko Chikondi

